# Bilateral septic arthritis of the sternoclavicular joint complicating infective endocarditis: a case report

**DOI:** 10.1186/s13256-018-1709-9

**Published:** 2018-07-05

**Authors:** Karim Masmoudi, Emna Elleuch, Rim Akrout, Afef Feki, Mariam Ezzeddine, Hela Fourati, Dorra Lahiani, Mounir Ben Jemaâ, Sofiène Baklouti

**Affiliations:** 1 Medicine Faculty of Sousse, Mohamed Karoui Avenue, zip code 4000 Sousse, Tunisia; 2 Infectious Diseases Department, Hédi Chaker University Hospital, El-Aïn Street Km 0,5, zip code 3029 Sfax, Tunisia; 3 Rheumatology Department, Hédi Chaker University Hospital, El-Aïn Street Km 0,5, zip code 3029 Sfax, Tunisia

**Keywords:** Sternoclavicular joint, Septic arthritis, Bilateral presentation, Infective endocarditis, Calcium pyrophosphate dihydrate crystal deposition disease, Case report

## Abstract

**Background:**

Septic arthritis is an infectious disease that commonly affects weight-bearing or proximal joints such as the knee and the hip. The sternoclavicular joint is an unusual site of this entity. It usually occurs in patients with diabetes mellitus, intravenous drug abusers, or those with rheumatoid arthritis. Analysis of the previous literature showed few articles and these described essentially cases of unilateral presentation.

**Case presentation:**

We report a rare case of a bilateral septic arthritis of the sternoclavicular joint sustained by a 71-year-old Tunisian woman whose medical history was significant for methicillin-resistant *Staphylococcus aureus* infective endocarditis 6 months ago. Imaging investigations revealed destruction of the medial extremities of her two clavicles and bilateral collections in the soft tissues around her sternoclavicular joints. She was treated successfully by needle aspiration drainage combined with a 12-week antibiotherapy*.*

**Conclusions:**

Bilateral septic arthritis of the sternoclavicular joint is an extremely rare entity, with a paucity of literature. Only early diagnosis, which is obtained from the culture of the joint fluid using needle aspiration, allows satisfactory functional outcome and a good prognosis*.*

Osteoarticular infections should be considered in patients with recent infective endocarditis in cases of fever recurrence*.*

## Background

Septic arthritis (SA) of the sternoclavicular joint (SCJ) is a rare condition, affecting only 1% of all joints [1–5]. Diagnostic delay has often been reported because of the insidious course of the infection, with no specific symptoms [1–6]_._ This uncommon location may affect healthy individuals [7], but it mainly occurs in patients with predisposing risk factors such as diabetes mellitus, hemodialysis, intravenous drug abuse, subclavian vein catheterism, and rheumatoid arthritis [1–3, 5–7]. SA of the SCJ is unilateral in 95% of the cases [1, 4, 6, 7]. We report a rare case of bilateral SA of the SCJ secondary to an infective endocarditis.

## Case presentation

A 71-year-old Tunisian woman presented to our emergency department with atraumatic pain in her neck and shoulders, and fever that had evolved over 4 weeks. Her medical history was significant for arterial hypertension and calcium pyrophosphate dihydrate deposition (CPDD) disease managed by non-steroidal anti-inflammatory drugs. She had no medical family history, and she had not undergone any surgical intervention. She also sustained, 6 months ago, an infective endocarditis due to methicillin-resistant *Staphylococcus aureus* (MRSA) that was successfully managed with medical treatment (an adapted 2-month antibiotherapy). Infective endocarditis was diagnosed by suggestive findings on transesophageal echocardiogram (irregular 10–15 mm vegetations attached to the aortic and mitral valves) and isolation of MRSA on two consecutive blood cultures. Since she had a moderate aortic and mitral regurgitation, no operative treatment was necessary according to our cardiothoracic surgery team. She was given intravenously administered antibiotics using a combination of vancomycin at 30 mg/kg per day for 8 weeks and gentamicin at 3 mg/kg per day for 5 days. No other blood cultures were performed since she was afebrile from the third week of antibiotherapy with a negative C-reactive protein (CRP) at the last week of antibiotherapy (Table 1).

**Table 1:** Timeline

	November 2, 2007	November 5, 2007	November 21, 2007	December 31, 2007	January 31, 2008	April 10, 2008	May 8, 2008	May 12, 2008	August 7, 2008	May 7, 2009
Time	T = 0	T = 3 days	T = 19 days	T ≈ 2 months	T ≈ 3 months	T ≈ 5 months	T = 6 months + 6 days	T = 6 months + 10 days	T ≈ 9 months	T ≈ 21 months
Event	Initial presentation: Infective endocarditis	1st day of adapted antibiotherapy: Vancomycin+ gentamicin	First day of apyrexia	End of antibiotherapy. Apyrexia CRP < 6 mg/l	1 month-follow-up. Apyrexia CRP = 0	Fever recurrence	Current presentation: Bilateral SA of the SCJ	1st day of adapted antibiotherapy	End of antibiotherapy	12 months follow-up of the current presentation

*CRP* C-reactive protein, *SA* septic arthritis, *SCJ* sternoclavicular joint

At the current presentation, a physical examination revealed a painful and tender swelling over her right SCJ, and the overlying skin was stretched and shiny without any productive sinus. Her rectal temperature was 39 °C. There was a moderate decrease in her right shoulder’s range of motion. Her cardiac auscultation did not reveal any added sounds or other abnormalities. Laboratory investigations showed an erythrocyte sedimentation rate of 107 mm at the end of 1 hour, and a CRP at 222 mg/l.

**Fig. 1:**
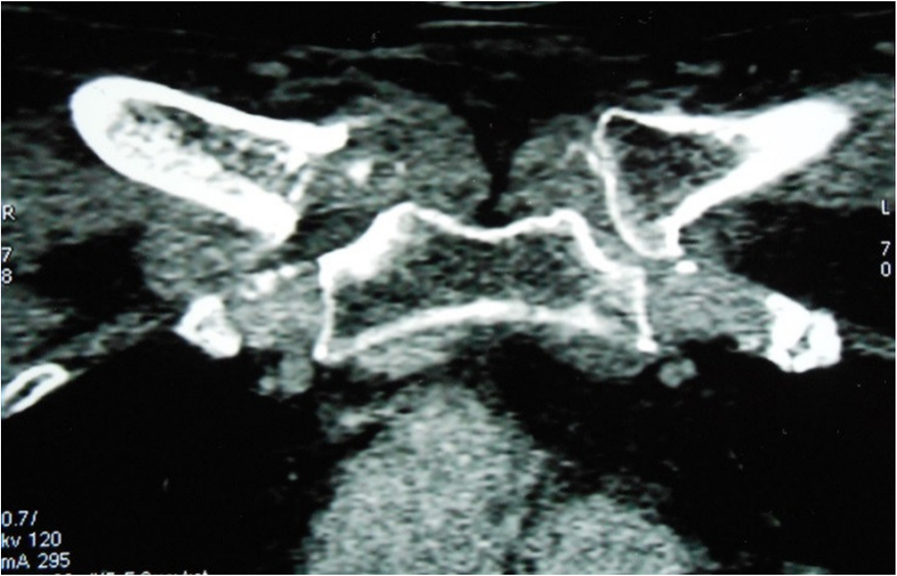
Computed tomography scans of the sternoclavicular joints demonstrating destruction of the medial extremities of the clavicles

**Fig. 2:**
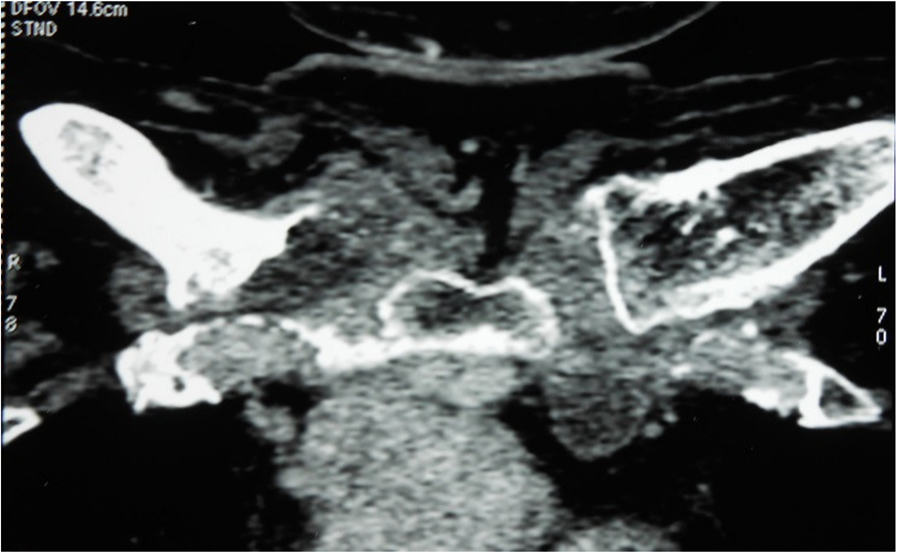
Computed tomography scans of the sternoclavicular joints showing bilateral soft tissue collections

**Fig. 3:**
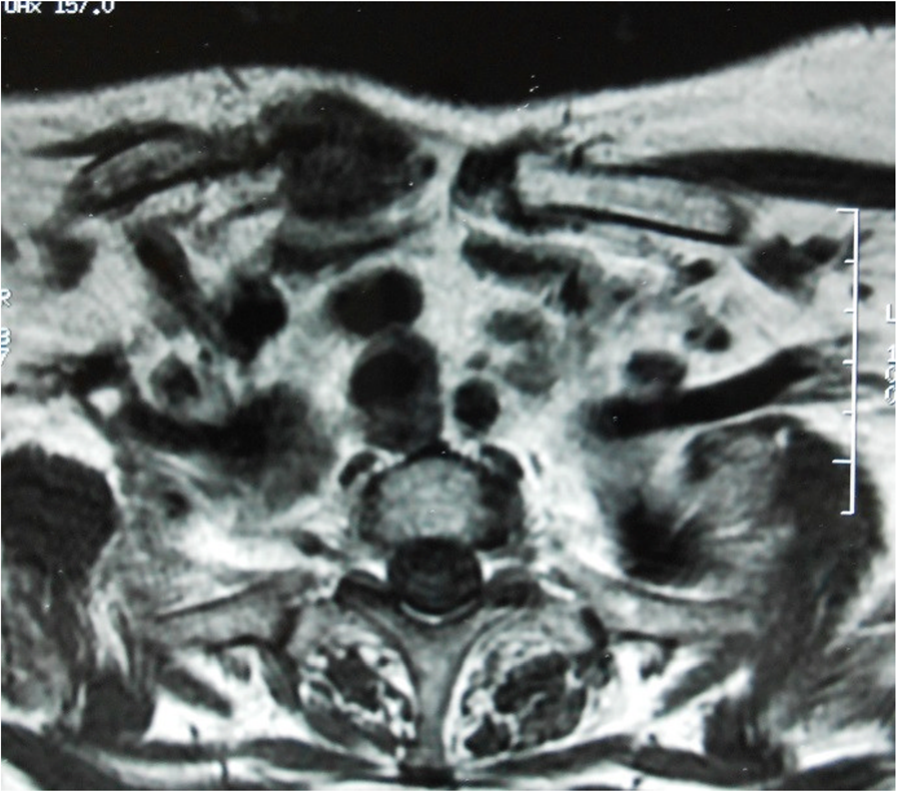
Magentic resonance imaging indicates subchondral edema of the medial extremities of the clavicles

**Fig. 4:**
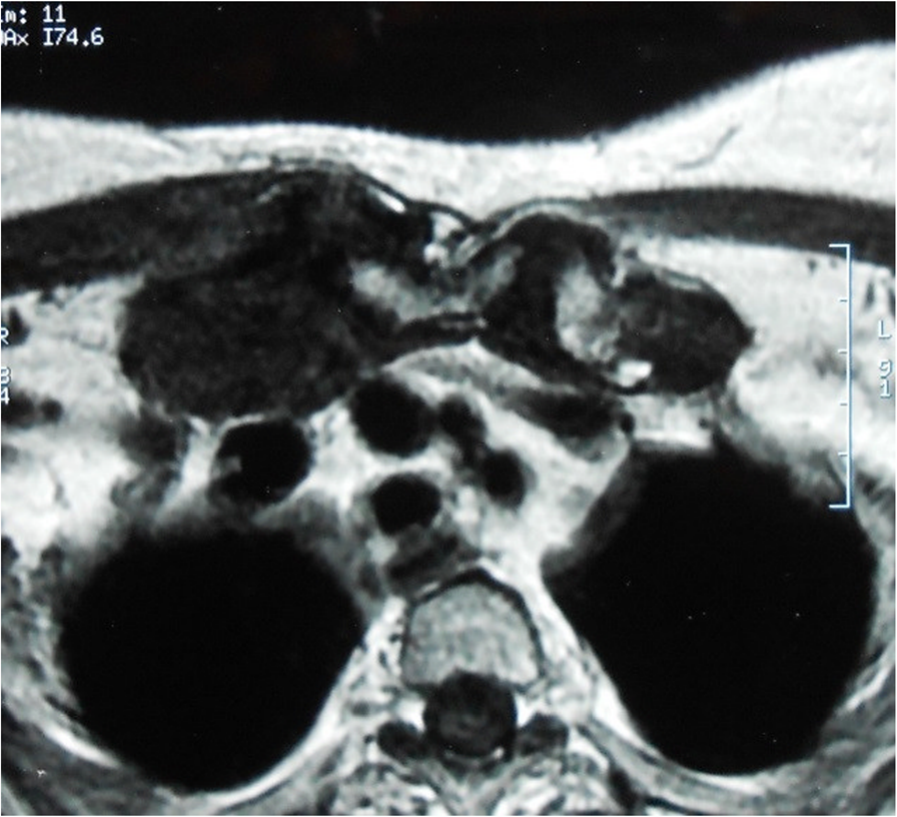
Magentic resonance imaging revealing soft tissue collections around the sternoclavicular joints

Computed tomography (CT) scans revealed a destruction of the medial extremities of her two clavicles and bilateral collections in the soft tissues around the SCJs (Figs. 1 and 2). Magnetic resonance imaging (MRI) showed an osteolysis of both sternal and clavicular margins of her SCJs, with a subchondral edema and soft tissue collections (larger on the right side; Figs. 3 and 4).

Fine-needle aspiration of her right SCJ fluid was performed as well as synovial biopsy. Blood and joint fluid culture were positive for MRSA*.* Histologic examination was significant for pyogenic SA.

Intravenous antibiotherapy using an association of teicoplanin and ciprofloxacin was administrated for 1 month, followed by orally administered antibiotherapy combining pristinamycin and ciprofloxacin for 2 months.

We noticed a complete resolution of her fever and the swelling without any biological or clinical signs of recurrence at the last follow-up of 12 months.

## Discussion

Unilateral SA of the SCJ is not exceptional. Literature analysis showed that bilateral presentation is scarcely published. Ross and Hala (2004) reported in a systematic review of 180 patients who sustained a SA of the SCJ, that only 8 patients (4%) had a bilateral localization [6]. To the best of our knowledge, only one case report describing a bilateral pyogenic SA of the SCJ was found. It was secondary to an infection of a central venous catheter [2].

The SCJ is usually seeded from systematic bacteremia [6]. This may explain the high incidence of this disease in particular populations: those who administer drugs intravenously, and patients with subclavicular or internal jugular vein catheter [2].

Osteoarticular infections secondary to infective endocarditis have been reported in several studies. Its incidence varies from 4.3 to 8.8% of all cases of infective endocarditis [8, 9].

In our case, the SCJ involvement was due to an infective endocarditis that occurred 6 months ago. The medical history of our patient was not significant for diabetes mellitus, hemodialysis, or central venous catheterism. However, she had some risk factors of SA (advanced age, recent history of a deep infection site, CPDD). The final diagnosis was quite challenging in this field because of her chronic and diffuse polyarthralgia due to the CPDD disease.

The clinical features of SA of the SCJ might be explained by the anatomic particularities of the SCJ: the capsule of this joint is reinforced by strong sternoclavicular ligaments and is consequently unable to distend [1, 2, 6]. The lack of prominent joint effusion may contribute to a delay in the presentation (the median duration of symptoms at presentation is much longer than the other sites of SA), as well as a propagation of the infection in deep soft tissues and the chest wall [3, 6].

Involvement of the SCJ in CPDD disease is extremely rare. In the previous literature, two cases have been reported. According to Borowski *et al*. (2015), there has been only one report of this unusual entity in addition to their study [10, 11].

Involvement of our patient’s left SCJ was discovered fortuitously, despite the evidence of an established infection on CT scan and MRI (bone destruction of her left clavicle, soft tissue collection). Moreover, a physical examination did not reveal any swelling or pain around her left SCJ. These findings confirm the paucity of symptoms in SA of the SCJ and its insidious onset [1–6]. They may also explain the potential seriousness of this entity. Consequently, SA of the SCJ is often diagnosed at late stages with locoregional or systemic complications (chest wall abscess in 25% and mediastinitis in 13% of the cases according to Ross and Hala) [1–3, 6, 7]. In this case, our patient sustained two locoregional complications: bone destruction and soft tissue collection.

Management of SA of the SCJ remains, to date, controversial with no therapeutic consensuses [5]_**.**_ It can be managed by non-operative methods. In Ross and Hala’s review, 58% of the patients (104 out of 180) required surgery, among which 10% were operated because of failure of the medical therapy [6]. Therefore, according to this study, isolated medical treatment was considered in 52% of the cases. Surgical debridement and joint resection are commonly indicated in cases of extensive bone destruction, mediastinitis, chest wall abscess, pleural involvement, retrosternal collection, and the necessity of surgical biopsy. The medical treatment may be successful in cases of limited disease revealed on radiological findings [1, 2, 6, 7].

Our patient did not undergo surgical debridement for multiples reasons:

- There was not any serious complication or chest involvement.
- Histologic examination, after fine-needle biopsy, was significant for pyogenic SA, and a surgical biopsy was not necessary.
- Bacteriological investigations were conclusive, with a culture-proven arthritis.
- Soft tissue collections were subcutaneous, superficial, and accessible to needle evacuation.

Given the involvement at different stages of the two SCJs, we think that the right side was affected first by a bacteremia, whereas the left one was infected, later, by contiguity from the right side. The invasion of the right SCJ could have been contemporary to the infective endocarditis but remained silent for 6 months, since *Staphylococcus aureus* is part of small colony variants of bacteria. Therefore, bacteria that were the same as those of the initial episode were isolated.

## Conclusions

SA of the SCJ with bilateral presentation is unusual and scarcely published in the literature. Its management involves surgical or needle drainage combined with parenteral antibiotherapy. SA of the SCJ can be successfully managed with medical treatment even in cases with locoregional complications, thanks to the advances in antibiotherapy and imaging techniques that optimize the results of fine-needle biopsy and guide the needle drainage.

The diagnosis of sternoclavicular SA should be considered in every case of unexplained neck or shoulder pain, even without local symptoms in the sternoclavicular area (swelling, erythema, skin abnormalities, and pain).

In spite of their rarity, bone and joints infections should be considered in patients with a history of infective endocarditis, in cases of fever recurrence.

## Abbreviations

CPDD: Calcium pyrophosphate dihydrate deposition
CRP: C-reactive protein
CT: Computed tomography
MRI: Magnetic resonance imaging
MRSA: Methicillin-resistant *Staphylococcus aureus*
SA: Septic arthritis
SCJ: Sternoclavicular joint

## Authors’ information

KM is a university hospital assistant in the orthopaedic department; AF is a senior resident in the rheumatology department. EE is an associate professor at the infectious diseases department.

ME is a consulting physician at the rheumatology department. SB is the head of the rheumatology department. MB is the head of the infectious diseases department. RA and HF are associate professors at the rheumatology department. DL is an associate professor at the infectious diseases department.
